# An Intervention to Connect Patients With Psychosis and Volunteers via Smartphone (the Phone Pal): Development Study

**DOI:** 10.2196/35086

**Published:** 2022-06-02

**Authors:** Mariana Pinto da Costa, Phone Pal Advisory Groups

**Affiliations:** 1 Institute of Psychiatry, Psychology & Neuroscience King's College London London United Kingdom; 2 Barts and The London School of Medicine and Dentistry Queen Mary University of London London United Kingdom; 3 Institute of Biomedical Sciences Abel Salazar University of Porto Porto Portugal

**Keywords:** intervention, intervention development, digital mental health, psychosis, severe mental illness, volunteering, volunteer, mental health, mental illness, development, design, user centered design, smartphone, mobile phone, mobile health, mHealth, MRC framework, Medical Research Council framework

## Abstract

**Background:**

Intervention development is a critical stage. However, evidence indicates that the substandard reporting of intervention details is widespread.

**Objective:**

This study aimed to provide an overview of the guiding frameworks, methodology, and stages for the design and construction of a new complex intervention—the Phone Pal.

**Methods:**

The intervention development process followed the Medical Research Council framework for developing complex interventions as well as the person-based approach. The intervention was developed following the evidence synthesis of a literature review, a focus group study, and a survey after consultation and input from advisory groups with a range of stakeholders, including patients, volunteers, clinicians, and academics.

**Results:**

The developed logic model outlines the contextual factors, intervention, mechanisms of change, and short- and long-term outcomes. The operationalized intervention required matching 1 patient with 1 volunteer to communicate with each other through a smartphone via SMS text messages, WhatsApp messages or email, and audio or video calls. Each participant was encouraged to communicate with their match at least once per week for a 12-week period using informal conversation.

**Conclusions:**

The systematic process and theoretically sound strategy through which this intervention was developed can provide insights to future researchers on the reality of developing and preparing the operationalization of a digital intervention using multiple components.

## Introduction

### Complex Interventions

The distinctive notion of a complex intervention has emerged since 2000 [1] through debate focused on the definitions of complex interventions and their components [2]. In particular, there is a need to identify the active ingredients [3], especially in multicomponent psychosocial interventions. It has been emphasized that the description of complex interventions is not enough [4]. In fact, the process of decision-making during research and whether relevant stakeholders are involved and how is often not described.

The 2010 CONSORT (Consolidated Standards of Reporting Trials) statement recommends that authors report interventions with “sufficient details to allow replication” [5]. However, evidence indicates that substandard reporting of intervention details is widespread [6,7], which may be due to the complexity of many nonpharmacological interventions carried out within a social context [4,8].

It has been recommended that complex interventions should be defined as being formed of parts; these may be material, human, theoretical, social, or procedural in nature and may themselves have subdivisions. These can be further stratified into higher and lower realms that exercise power individually, in combination, or as emergent properties [9]. The intervention *whole* refers to the intervention as a single complete entity distinct from the parts that comprise it; its existence depends on its parts. However, some approaches view the whole as being more than the component parts; thus, the parts are insufficient to explain changes in the intervention’s outcomes [10]. Intervention developers routinely elicit the views of target users in a variety of ways [11,12]. However, there is still controversy surrounding how best to do this [13].

### The Phone Pal as a Case Study

Volunteering programs appear beneficial and can be encouraged as a means of integrating patients with severe mental illness (SMI) into their communities and promoting their recovery [14]. SMI typically refers to someone with a diagnosis of psychosis of >2 years, duration which impairs their functionality [15].

Social relationships constitute a complex and multidimensional construct with both structural and functional components. With respect to patients with psychosis, there is a body of literature describing their social isolation, low quality of life, low self-esteem, sedentary lifestyle, and nonsecure attachments [16-20]. Regarding volunteers, studies have suggested improvements in their quality of life and changes in their attitudes toward people with mental illness [21].

In the current era of innovation, new models of volunteering may arise using technology. These may encourage the recruitment of new kinds of volunteers and also open the possibility of remote volunteering across large distances [22]. Thus, there is an additional need for remote models that enable volunteer support using technology to connect people with mental illness to others [23].

In an empowerment model in which volunteers take an *active* role as proactive citizens (eg, by supporting either a neighbor or someone remotely), digital volunteering could be an invaluable public health resource for society [24]. Having a Phone Pal as an intervention establishing informal communication about daily life could provide a distinctive form of mental and social support to people with mental illness, improving their mental and physical health [25].

### Objectives

The aim of this study was to report the process of development of a complex intervention (ie, the development of a logic model and the planning of its operationalization) as well as to report on the resultant intervention that was ultimately developed—the Phone Pal.

## Methods

### Overview

The process of developing the multicomponent behavioral intervention was performed systematically in different stages. It involved three aspects: (1) developing the logic model, (2) outlining the intervention components, and (3) operationalizing the intervention.

A logic model is a diagrammatic representation of an intervention describing anticipated delivery mechanisms (ie, how resources will be applied to ensure implementation), intervention components (ie, what is to be implemented), hypothesized mechanisms of impact (ie, how an intervention will work), and intended outcomes [26]. Logic models are commonly used to represent the causal processes through which interventions produce outcomes [27].

**Figure 1:**
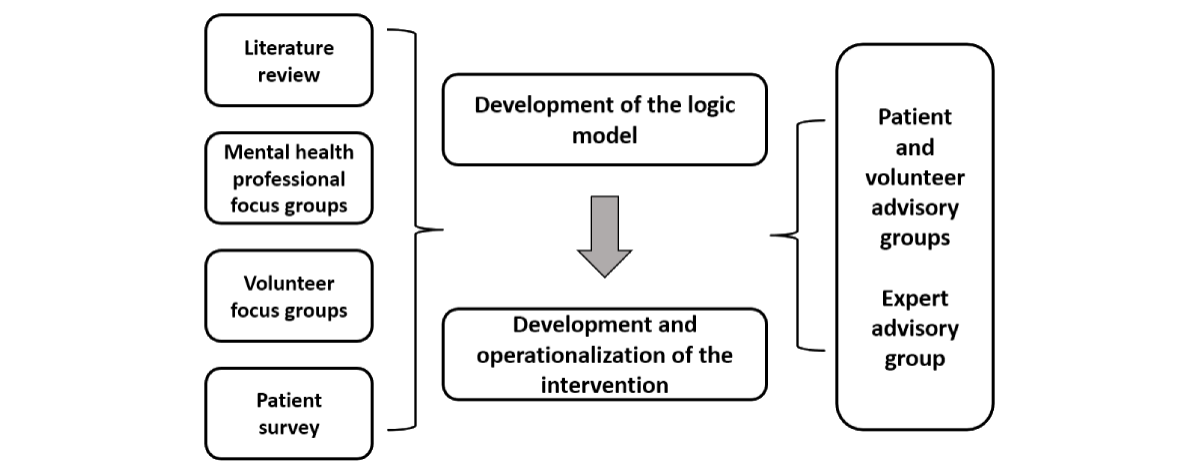
Framework of methods and stages for the intervention development and testing.

The development of the Phone Pal logic model followed the recommendations of the 2 guiding frameworks for this study: the UK Medical Research Council (MRC) framework for developing and evaluating complex interventions [28] and the person-based approach [29]. Both frameworks specify the importance of illustrating the theoretical processes that are expected within an intervention and its context. Figure 1 illustrates the various stages of the development pathway followed in this study.

### Guiding Frameworks

The MRC framework [28] is the most widely used framework for intervention development with multiple components. The MRC framework has four interlinked stages: (1) development, (2) feasibility and piloting, (3) evaluation, and (4) implementation.

This study focuses on the first stage (ie, development), which entails the assessment of the existing evidence base followed by the identification and construction of the theory relating to an intervention. This was relevant to this study, as the mechanisms of volunteering remain unclear.

The person-based approach was selected as a supplementary framework for intervention development [29]. This approach recognizes the specific contextual challenges related to engaging users with digital interventions designed for independent use. This framework argues that interventions must be appealing, easy to use, and relevant to the participants’ needs; otherwise, people will not use them.

The person-based approach also advocates that the conceptual modeling described in the MRC framework should consider specific contextual behavioral issues and challenges identified during intervention development. The creation of guiding principles is advised to address contextual challenges and inform an intervention logic model. There are two elements: (1) intervention objectives and (2) core components of the intervention that operationalize the objectives. This approach puts emphasis on component design to improve digital health intervention acceptability and engagement and suggests that all interventions should aim to promote a positive emotional experience [29].

### Stages of the Intervention Development

The 4 stages of intervention development used in this study were based on the MRC framework and the person-based approach [28,29] (Multimedia Appendix 1).

### Methods Involved in the Intervention Development

#### Overview

Different methods were used in the intervention development. Some of these activities took place concurrently (eg, the focus group study and survey) and were followed by several iterations of consultations with the advisory groups. The review was conducted between October 2015 and December 2015, the focus groups were conducted between January 2016 and September 2017, the survey was conducted between August 2016 and August 2017, and the advisory groups were consulted from February 2017 to February 2020.

#### Review of the Literature

A rapid narrative literature review was conducted to enable efficient mapping of the main results related to the specified field. It constitutes a useful technique for intervention development where a broad perspective of the literature is required within a limited timetable [30,31]. Key papers and theories for developing interventions and digital interventions were subsequently searched [32].

#### Focus Group Study

Stakeholders potentially linked to the provision of volunteering (ie, mental health professionals and volunteers) were interviewed in a focus group format to explore their views regarding the relationship formats between patients and volunteers [32]. A total of 24 focus groups were conducted with 119 participants. The process and findings of the focus groups have been described elsewhere [33,34].

#### Survey

Patients’ preferences with respect to contact with a volunteer were assessed through a survey that evaluated potential relationship formats between patients and volunteers and the patients’ preferred volunteer characteristics [22]. A total of 151 patients with psychosis followed in outpatient services in London were interviewed. The findings of this study have been published elsewhere [22].

#### Volunteer and Patient Advisory Groups

In this research, both patients and volunteers were also included as patient and public involvement advisors [35]. The lead author (MPdC) worked with organizations of volunteers and patients consulting them regarding intervention development [36,37].

The lead author collaborated with a national volunteering association, Befriending Networks [38], which is the umbrella charity for befriending services operating across the United Kingdom and beyond. In September 2017, the lead author organized an event in London that brought several volunteer organizations and volunteers together. The event raised awareness of research on volunteering in mental health and was attended by volunteers involved in charities of mental health. Many of these volunteers contributed to the focus groups conducted during this event. Following the meeting, 2 volunteers also worked with the lead author through the advisory group offering their input for the intervention development process.

To establish patient views, the lead author worked with the Service User and Carer Group Advising on Research at City University of London [39]. This group comprised 14 mental health service users who had personal experience with a variety of mental health conditions, including psychosis.

These groups were consulted throughout the intervention development phase; the meetings involved discussions based on written materials or presentations. The opinions of potential users (ie, both patients and volunteers) were sought concerning which components they thought should be included in an intervention that was aimed toward the provision of opportunities for volunteering in mental health.

The Phone Pal logic model was then developed in consultation with the advisory groups, and it explored a set of person-based intervention components and the potential outcomes that the intervention could affect aligned with the participants’ opinions on what to achieve. The groups advised on the suitability of different components and provided recommendations on the role and characteristics of patients and volunteers in this intervention.

These potential users offered important insights to establish the guiding principles of the intervention. They also discussed and specified the key components and objectives that would improve acceptability and engagement with a digital intervention.

#### Expert Advisory and Consultation Group

Regular meetings with a multidisciplinary team of 30 people comprising principal investigators, postdoctoral researchers, PhD students, researchers, public health experts, anthropologists, psychologists, and psychiatrists at the Unit for Social and Community Psychiatry were used to discuss the intervention development process. The lead author presented the guiding principles, and the team advised on the Phone Pal logic model development.

#### Evidence Synthesis

The findings emerging from the aforementioned methods were synthesized and presented to the advisory groups. Although there were divergent views on occasions, these group discussions frequently allowed one view to emerge as preferred or, on some matters, there was even consensus from the advisory group members. Further to the consultation with the advisory groups, actions were agreed upon to guide the design of key components of the intervention, the development of the logic model, and the intervention operationalization. The decisions that addressed the identified challenges were acceptable to patients and volunteers, were informed by theory, could be achieved pragmatically within this research, and were endorsed by the expert group.

### Ethics Approval

The initial intervention developed was assessed, and changes were requested by the Research Ethics Committee (REC). The final intervention was approved by the REC and the Health Research Authority in the United Kingdom (REC reference 18/EE/0196; protocol 012393).

## Results

### The Development of the Phone Pal Logic Model

#### Process for Selecting the Intervention Core Components and Objectives

**Figure 2:**
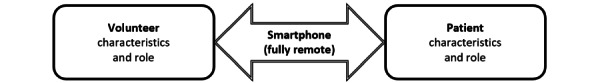
Core components of the intervention.

The process for selecting the intervention core components and objectives was guided by the intervention guiding principles. The guiding principles of the intervention communicate how the objectives and components of the intervention address the particular contextual challenges identified during the process. After an extensive process of gathering the existing evidence, three core intervention components were outlined: (1) a *match* (ie, patient-volunteer), (2) remote communication, and (3) a smartphone, as depicted in Figure 2.

The objectives established were (1) to provide one-to-one access to a *match*, (2) to provide this access to a person remotely, and (3) to allow the communication to occur digitally. Table 1 presents the core components, intervention objectives, and contextual factors.

The following section describes the process of logic model development, portraying the decision-making for the selection of suitable intervention components—namely, the volunteers’ characteristics and role, the patients’ characteristics and role, and being connected remotely and through a smartphone—using the methods that informed them (Table 2).

**Table 1:** Core components, intervention objectives, and contextual factors.

Core components	Intervention objectives	Contextual factors
A “match”	Provide one-to-one access to another person—a match	Patients might be socially isolated
Remote communication	Enable the communication to occur remotely	Patients might face barriers to in-person meetings
A smartphone	Provide access to a person digitally	Technology is integrated into people’s lives

**Table 2:** Components, descriptions, and methods.

Component	Brief description	Literature review	Focus group study	Survey	Patient and volunteer advisory groups	Expert advisory group
Volunteer characteristics	To be inclusive in the definition of volunteers (ie, recruit from a variety of backgrounds and include people with and without personal mental health experience)	✓	✓	✓	✓	
Volunteer role	To provide human contact to patients, establishing informal communication between each other about daily life as part of a more symmetrical relationship	✓	✓	✓	✓	✓
Patient characteristics	To focus on patients with psychosis who are usually the most socially isolated group	✓			✓	✓
Patient role	To obtain human contact from volunteers, establishing informal communication between each other about daily life as part of a more symmetrical relationship	✓	✓		✓	✓
Fully remote	To encourage participants to only communicate with each other remotely	✓				✓
Smartphone	To encourage that participants communicate with each other through a smartphone	✓	✓	✓	✓	

#### Components

##### Volunteer Characteristics

Volunteers have varied profiles. It was concluded from systematic reviews [40,41] that there is no *typical* volunteer. These variations [42] encouraged the adoption of a definition of diverse volunteers for the inclusion criteria of this study that enabled volunteer recruitment from a variety of backgrounds.

The volunteer inclusion criteria were widened, recruiting volunteers without experience of mental illness. Although peer support is a theoretical intervention that has been used in web-based psychological interventions to promote co-operation and expertise and reduce loneliness [43], it excludes volunteers who do not possess a shared characteristic.

Volunteers with personal experience of mental illness were also included in this study. Several articles document volunteers who disclose a personal psychiatric history themselves [44-46]. Such volunteers can act as role models and be an inspiration to those with a current mental illness as they are able to demonstrate that *life does go on* and that it is possible to cope with an SMI [44].

In addition, the patient survey demonstrated that most patients (83/148, 56.1%) were interested in a volunteer who had personal experience as a patient in mental health care [22]; these findings added weight to the decision to broaden the inclusion criteria. The focus group study also presented a range of qualities and characteristics related to volunteers. However, it did not advocate for any specific *profile* of volunteer; views ranged from *anyone can be a volunteer* to those who considered that individuals with a *supporting* profile and skills should be the ones providing volunteering support.

For the new intervention, the literature reinforced the decision to have broad inclusion criteria for volunteers, which could encompass a wide range of individuals with or without a possible history of mental issues. The patient and volunteer advisory groups endorsed this choice.

##### Role of the Volunteer

There are several variations in the potential role of volunteers described in the literature. Although overall these are referred to as *social support* [47], such an approach may comprise emotional, informational, appraisal, and instrumental aspects [48]. Social support has been described as a reciprocal process, where its provision may be as important as its receipt [49].

In the focus group study, stakeholders expressed strong views that the intervention should aim to overcome isolation by connecting patients to additional social contacts, thus using volunteers as *instruments* for *modeling* (ie, as a transition figure).

Of the surveyed patients interested in technology, most (32/56, 57%) thought that the aim of digital volunteering was to make a new friend and had less to do with increasing their activities. They appeared to view technology as a means of establishing contacts with other people and forming friendships. The fact that most of these patients wanted someone as a volunteer with personal experience of mental illness implied a desire to have a more symmetrical relationship.

Some of the members of the expert advisory group initially advised that the volunteers should maintain an *asymmetrical* role of patient support. However, in view of the feedback collected from the focus group study and the survey, most of the expert group members subsequently supported the pursuit of a new model in which a more *symmetrical* relationship was recommended between the 2 matched participants (ie, one that followed the guiding principles of social facilitation, social learning, social role, social comparison, and self-determination).

Encouraging relationship symmetry was a significant decision, distinguishing the current model from more traditional frameworks of befriending or peer support, which exclude people with or without mental illness, respectively. In this intervention, both were accepted as volunteers. In addition, it was deemed important to incorporate the assessment of social contacts as one of the study outcomes before and after the intervention.

With the endorsement of the patient and volunteer advisory groups, it was decided that the primary role of the volunteer in this intervention would be to offer remote human contact to patients, establishing informal communication between each other about daily life. The volunteer could be a potential role model for the patient, establishing a more symmetrical relationship. Therefore, the focus of this intervention is the patient’s and volunteer’s social interactions with each other via smartphone as opposed to being centered on being together and engaging in social activities.

##### Patient Characteristics and Role

The literature describes that, in people with SMI, social isolation has been linked to higher levels of delusions [50], lack of insight [51], and high hospital use [52]. Importantly, previous studies have found a significant association between loneliness and psychosis [53]. The significance of social isolation in patients with psychosis led to the decision to focus this intervention on these individuals.

The focus group study provided insights into the role of patients when outlining the character of the relationships between patients and volunteers.

The survey revealed that younger patients and those with a more recent diagnosis of psychosis were more likely to prefer digital volunteering [22]. However, another study reported that older patients with psychosis were actually more likely to engage with digital interventions [54]. In light of these data, it was opted to include adult patients of all ages in the study. This would also enable the comparison of intervention use according to age. The advisory groups endorsed these proposals for the patients’ characteristics and their role in this intervention.

##### Connected Remotely Through a Smartphone

The concept of volunteers and patients interacting through technology is reinforced in the literature. Technology can help connect people, especially those usually hard to reach [55]. It facilitates more frequent and flexible communication, which is the central element in a relationship. In particular, smartphones are highly portable and in widespread use, presenting new opportunities to deliver interventions, monitor behavior changes, and make communication easier between people who are far away from each other [56-58].

It has been documented that patients with psychosis value technology [59]; are interested in using smartphones [60]; and have been found to engage, adhere, and be satisfied with their use [61-63]. In fact, people with SMI were able to use and adhere to digital interventions [64] even when experiencing negative symptoms [65] and generally found such technology helpful and easy to use. A study evaluating a digital intervention in people with schizophrenia reported that noncompleters were more likely to have severe negative symptoms than completers but found no difference in the incidence of positive or depressive symptoms [66].

People have long established contact with strangers even before technology was in place. This is illustrated by the popularity of *pen pal* letters abroad [67]. This concept supported the decision to design a fully digital intervention and not encourage each match to meet in person during the study.

Among the array of digital tools currently available, smartphones were favored rather than alternatives such as a website or tablet. For the former option, the participants would need constant internet access; with respect to the latter, tablets are commonly larger and less portable than smartphones. The plethora of evidence relating to smartphone use, ownership, and interest in people with psychosis also influenced the decision to deliver the intervention via this modality [59-65]. It was also the most affordable option to enable remote communication between patients and volunteers.

The results from the patient survey revealed that several patients (56/151, 37.1%) were interested in receiving digital volunteering; only a few patients (20/151, 13.2%) did not use technology.

A further important consideration in relation to smartphone use was the advice from the focus group study that veered away from the idea of a separate app. An app designed specifically for people with mental illness to communicate with *healthy* volunteers could potentially risk a further increase in stigma. Instead, a strong recommendation was made to use the normal modalities of communication typically offered by every smartphone (eg, audio calls, video calls, written messages, and emails).

The expert advisory group endorsed the use of this totally remote style of communication to differentiate from face-to-face interactions (Multimedia Appendix 2).

### Process for Selecting the Guiding Principles for the Phone Pal Logic Model

#### Overview

The guiding principles aim to address the contextual challenges that are likely to affect intervention delivery. They consist of 2 aspects (ie, the intervention objectives and the core components used to operationalize them), which were developed to ensure that the intervention met the volunteers’ and patients’ expectations [68].

This logic model was assembled with the contextual factors and anticipated outcomes following a process of collating existing evidence, varying theories, and conceptualizations. Table 3 illustrates the guiding principles of the intervention, hypothesized mechanisms of change, and outcomes that may be affected by intervention use. The intervention components were selected after feedback to theoretically promote engagement via role modeling and self-efficacy, as further explained.

**Table 3:** Guiding principles, mechanisms of change, and outcomes.

Guiding principles	Mechanisms of change	Patient outcomes	Volunteer outcomes
Personalization, tailoring, and real-world feel	Engagement	Symptoms and physical activity	Physical activity
Social facilitation, social learning, social role, social comparison, and normative influence	Role model	Social contacts and social comparison	Social distance and social comparison
Self-monitoring, co-operation, and recognition	Self-efficacy	Self-esteem, attachments, and quality of life	Self-esteem and quality of life

#### Guiding Principles

Different guiding principles were then grouped as drivers of the 3 distinct mechanisms of change [68].

A total of 3 guiding principles were linked with *engagement* as a mechanism of change (ie, *personalization*, *tailoring,* and *real-world feel*). *Personalization* entails the offer of personalized content; *tailoring* encompasses the adaptation of the content to potential needs, interests, personality, context, or other individual factors; and the *real-world feel* emphasizes to the researcher or responsible organization the need to increase the intervention credibility.

In total, 5 guiding principles under the auspice of *role model* form the second grouping (ie, *social facilitation*, *social learning*, *social role*, *social comparison*, and *normative influence*). *Social facilitation* postulates that participants are more likely to perform a target behavior if they are aware that another person is performing the behavior along with them. *Social learning* describes that a participant will be more motivated to perform a target behavior if they are able to observe others performing the behavior. *Social role* suggests that participants will be more likely to perform a target behavior if an intervention adopts a social role. *Social comparison* describes that participants will have a greater motivation to perform a target behavior if they can compare their performance with that of others. *Normative influence* describes that an intervention can provide peer pressure to increase the likelihood that a participant will adopt a target behavior.

The 3 remaining guiding principles were linked with the mechanism of change of *self-efficacy*: *self-monitoring*, *co-operation*, and *recognition*. *Self-monitoring* postulates that an intervention that allows a participant to keep track of their own performance or status supports the participant to achieve their goals. *Co-operation* describes that an intervention can motivate participants to adopt a target behavior by leveraging humans’ natural drive to co-operate. *Recognition* indicates that, by offering public recognition for a participant, an intervention can increase the likelihood that the participant will adopt the target behavior.

#### Mechanisms of Change

The 3 mechanisms of change hypothesized in this model were engagement, role model, and self-efficacy; these are described in the following sections.

#### Engagement

There are different types of engagement (eg, active or passive), which are accompanied by a heterogeneity of overarching definitions and measurements [69]. The challenge of engagement has been described as one of the main issues with digital interventions [70]. Typically, initial levels of enthusiasm are only maintained for a period after which diminution of use ensues, especially when interventions are used in the *real* world [71]. This may be a problem given that most interventions are designed to become effective over a specific duration. In health care, motivations for compliance can be driven by perceived usefulness and achieving goals; these can facilitate the creation of habits [72].

The behavioral activation theory states that, without specific training, activation consists primarily of the scheduling of pleasant activities [73]. This was key for this intervention. An important objective was that participants were motivated by the intervention and considered it relevant to improving their condition and social context. This study aimed to have the patients engaged with the intervention, interacting with a *real* person—a community volunteer—who would be available to communicate with them and support them.

#### Role Model

A key underlying theory for this intervention is the weak ties theory, which postulates the importance of less strong ties such as acquaintances to have access to new information outside the core network [74]. A series of principles related to *social support* have been proposed in the Oinas-Kukkonen and Harjumaa [68] framework (ie, social learning, social comparison, and social facilitation, which involve some form of connection with others through the technology). The principles of social learning and social comparison require that the person be aware of what the other person is doing and their progress, potentially achieved by mutual sharing of achievements or witnessing them.

The intervention aimed to provide patients with access to another person (ie, a volunteer) who, through communication, could act as a role model. Theories on *role model* use have advocated that it can be a way of motivating people to perform novel behaviors and inspire them to set ambitious goals [75].

Although the role of volunteers has been described as to *be there* or *do things*, this view may overlap with the conceptualizations of taking a more *passive* or *active* role [14]. In this intervention, it was envisioned that a volunteer would take a somewhat active role realized through being available to communicate with the patient; participating in mutual encouragement to engage in social and physical activities; and aiming to improve their self-esteem, social contacts, and physical activity. The rationale was to motivate participants to choose and communicate through methods that suited them while remaining in contact with their usual friends, thus linking the intervention to their social world.

#### Self-efficacy

Typically, users of digital interventions must feel motivated and confident to independently use them. Therefore, self-determination theory [76,77] is particularly relevant to understanding how users respond to this intervention and how it differs for patients and volunteers.

Therefore, the intervention components were organized under 3 objectives relevant to the constructs of self-determination theory. This postulates that intrinsic motivation to engage in health behavior change will be enhanced by (1) supporting users’ need for autonomy and feeling self-directed; (2) increasing users’ sense of competence, control, and confidence; and (3) enhancing users’ perceived relatedness or support from the intervention.

Having a chronic mental illness (eg, psychosis) presents several challenges that create a barrier to self-efficacy and affect life quality. Self-esteem has been linked to an intrapersonal influence on self-efficacy, suggesting that increasing self-esteem may subsequently increase self-efficacy [78].

In this study, there were several decisions made to design the intervention with the objective of improving self-efficacy. This entailed the provision of 1 volunteer for each patient with whom they were matched and with whom they could communicate remotely. This allowed them to help their match and to be helped, thus improving their self-esteem and quality of life. Having an attachment to their match could enable a normative influence, co-operation, and recognition. Furthermore, to raise awareness of their physical activity, participants were also encouraged by the researchers to use an app, *Accupedo*, to check their step count, a further means to enhance their self-efficacy [79,80].

### Phone Pal Logic Model With Intervention Contextual Factors and Outcomes

#### Overview

**Figure 3:**
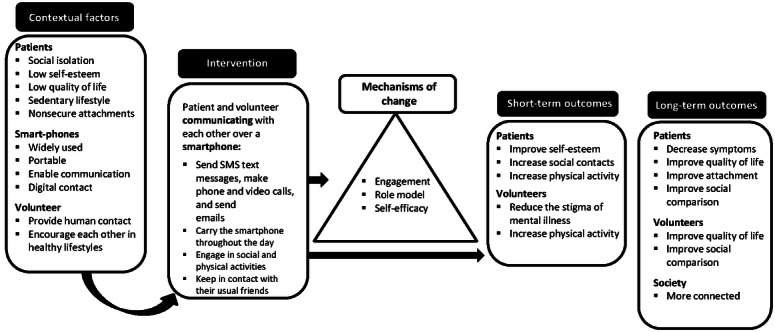
Logic model with the intervention contextual factors, processes, and outcomes.

Figure 3 depicts the Phone Pal logic model together with the contextual factors and anticipated outcomes. The model embraces the different components that were selected, the guiding principles promoted, the hypothesized mechanisms of change, and how these concepts are linked to outcomes. This logic model describes the main theoretical pathways connecting the components, principles, mechanisms, and outcomes of this intervention. In reality, the various intervention components may operate on multiple principles and, therefore, multiple mechanisms. An explanation follows for the rationale of why particular components and objectives were included in this intervention.

#### Contextual Factors

The Phone Pal logic model encompasses the contextual factors on which the intervention has been based: (1) patients’ social isolation, low self-esteem, low quality of life, sedentary lifestyle, and nonsecure attachments; (2) smartphone features (ie, wide availability, portability, and ability to offer digital and remote modes of communication); and (3) community volunteers who provide human contact with others, establishing informal communication about daily life, and offer support, being a role model to patients.

#### Short-term and Long-term Outcomes

In total, 2 types of outcomes were hypothesized for both patients and volunteers. These were classified according to the time frame (ie, short-term and long-term outcomes).

For patients, potential short-term outcomes were to improve their self-esteem, social contacts, and physical activity. In the longer term, it was felt that additional outcomes could be attained (ie, improving their quality of life, attachment, social comparison, and symptom alleviation).

For volunteers, the short-term outcome goals were to witness a change in their attitudes, with a decrease in their social stigma toward people with mental illness, and to increase their physical activity. Further outcomes could be achieved over a longer timescale (eg, improvement in quality of life and social comparison).

For both patients and volunteers, it was hypothesized that, through a higher level of engagement, they would become more *activated* and possibly increase their physical activity.

#### Phone Pal Intervention

The logic model of the planned intervention conceptualized the matching of 1 patient with 1 volunteer to communicate with each other over a smartphone. The core elements were (1) the remote medium for delivery of the intervention, (2) being in contact with another person, and (3) the use of a smartphone.

### Developing and Operationalizing the Phone Pal Intervention

#### Overview

*Core components* are intrinsically linked with the intervention basis; on a higher level are components that, although less fundamental than the core ones, are still part of the operationalized and envisioned intervention.

The main components describing how the intervention should be and the methods that informed them are summarized in Table 4. The methods and decisions made to operationalize the intervention are summarized in Table 5.

**Table 4:** Components of the intervention and the methods that informed them.

Component	Brief description	Literature review	Qualitative study	Survey	Patient and volunteer advisory groups	Expert advisory group
Communication duration	To encourage participants to communicate with each other for 12 weeks	✓			✓	
Communication frequency	To encourage participants to communicate with each other at least once per week	✓		✓	✓	
Communication methods	To encourage participants to communicate through the different methods that a smartphone provides (ie, sending messages or emails or making audio or video calls)	✓	✓	✓	✓	✓
Communication content	To encourage participants to have informal communication about daily life		✓		✓	✓

**Table 5:** Subcomponents of the intervention and the methods that informed them.

Component	Brief description	Literature review	Qualitative study	Survey	Patient and volunteer advisory groups	Expert advisory group
Matching	To match 1 patient with 1 volunteer to communicate with each other during the study	✓			✓	
Volunteer training	To provide training on communication, the boundaries of the relationship, and safeguarding	✓	✓		✓	✓
Patient training	To provide training on communication and the boundaries of the relationship	✓			✓	
Study coordinator	To have a study coordinator available to participants throughout the study offering access to support and supervision				✓	✓
Monitor communication	To monitor the communication of the participants through the offered smartphone				✓	✓
Monitor physical activity	To monitor the step count of the participants through an app on the offered smartphone				✓	✓

#### Duration, Frequency, and Methods of Communication

For pragmatic reasons, a period of 3 months was chosen in line with several publications reporting interventions with a duration of 12 weeks [54,81-86]. A review identified that the level of commitment should be for a minimum of 4 hours per month [44].

The idea of the communication being tailored by each participant is deemed important to create a persuasive system [68]. Therefore, in this intervention, it was decided to leave it up to each pair to decide which communication modalities they would like to use.

In young adults with a first psychotic episode, it was noted that they preferred a combination of technologies through which to receive mental health care (eg, SMS text message, video, and audio). Among these options, SMS text messages were preferred [87].

Surveyed patients [22] proposed that contacts should be open-ended and weekly and favored SMS text messages followed by email, Skype, Facebook, and audio calls. The modality *audio calls*, which was not given as a separate option in the survey, was named by different patients when selecting the choice *other*. The methods of communication initially selected for this intervention (ie, audio calls, video calls, SMS text messages, Facebook messages, WhatsApp messages, and emails) were determined according to the results of this survey and the literature. However, Facebook messages were removed following the instructions of the REC.

It was decided to encourage participants to communicate with each other at least once per week. The participants were informed that one of the study aims was to assess how much or how little each pairing decided to communicate. The focus group study findings indicated that people varied in their attitudes toward specific communication methods. In line with the person-based approach, it was decided not to be prescriptive in this regard.

A pragmatic decision was made to give the participants full access to and choice of all possible intervention components (ie, they could choose from sending SMS text messages, WhatsApp messages, or emails or making audio and video calls). The participants could opt to interact with their match through a particular tool, and their paired match could then choose to reciprocate through the same tool or select another modality. Relinquishing control of communication modality use has been supported by previous research [88]. The lead author encouraged volunteers to take the initiative to call patients in the first instance.

The expert advisory group supported the choice of communication method as all the tools were commonly accessible on any smartphone.

While developing the intervention, the lead author was prescriptive in the duration and frequency of the communication but not in the communication modalities. In the process of operationalizing the intervention, the lead author embraced the challenge of how much or how little guidance to provide when researching a psychosocial intervention. In terms of guidance for intervention adherence and use, there can be tension between supporting autonomy while still providing clear guidance on how participants can best change their behavior. Some studies reported concerns that offering too many options can be overwhelming [89]. Bestowing complete control can result in lower intervention use than *tunnelling* core intervention content [90]. Some researchers suggest that tunneling is less overwhelming than allowing patients free choice [68], and it is often used in mental health interventions [91]. However, a *tunnelled* approach [68] in which participants are led sequentially through intervention content, usually in a predefined order or based on a needs assessment, might not always be suitable and was deemed as too imposing. In fact, the person-based approach recommends that, in general, digital interventions should aim to promote user autonomy and offer choices where possible [29].

#### Communication Content

In this study, communication was between 2 humans and mediated through technology—the smartphone.

The focus groups provided insights into possible suggested conversation content for this intervention. The participants were instructed to establish informal communication about daily life.

#### Matching Patients and Volunteers

##### Overview

With respect to matching, previous research on telephone peer support has matched dyads randomly [49]; it was not possible to predict which dyad members would become friends based on interviewer impressions or similarity of individual characteristics. In the Volunteering in Mental Health befriending face-to-face trial, each patient-volunteer pairing was matched based on the instincts of the volunteer coordinator [92]. In the Phone Pal study intervention, it was decided to match on a first-come, first-served basis; the patient and volunteer advisory groups agreed. Specifically, personal characteristics or preferred communication methods were not used to influence this process.

##### Training Volunteers

The literature reports that volunteer training is compulsory in most programs [44,46,93-96], although some volunteers receive no training [97]. What this potentially means for volunteers varies, from not sharing personal contact details or information to, alternatively, sharing personal information and introducing the patient to friends or family members [96]. Examples of topics covered include expectations and responsibilities of a volunteer, preparation for managing initial meetings, general listening skills, boundaries and guidelines, mental illness, stigma, major diagnoses and symptoms, and conflict management [44,46]. The training offered to volunteers was primarily focused on communication and managing boundaries rather than presenting surplus information about the illness or treatment. The latter was important to avoid the concerns of *medicalizing* or providing unnecessary clinical information, which could jeopardize the establishment of a friendly relationship.

The focus group study provided a variety of views on the advantages and disadvantages of training. Although it was deemed important that training should be provided, there was no clear consensus; some thought it unnecessary. Concerns were also expressed about potentially high expectations, overinvolvement or risk of professionalizing volunteers, and breaching confidentiality. It was advised that these issues should be addressed with adequate training and also by asking volunteers to sign a confidentiality agreement, a common practice in many volunteer programs and research studies. This covered the requirement of volunteers not to disclose information about their matched patient to third-party agencies, friends or family, or anyone outside the research team without consent from the matched patient. In addition, they were requested not to disclose unnecessary information about their volunteer role to family, friends, or colleagues. Breaching confidentiality would only be permitted if there was a risk of serious harm (eg, if a patient expressed planned criminal intent against any individual or intent with a plan to commit suicide or self-harm; if a patient was judged to be at risk of sexual, emotional, or physical abuse; or where not acting on information would increase physical or emotional risk).

##### Training Patients

Upon the request of the REC, customized training materials were developed for patients; these were adapted from the materials developed for volunteers. The lead author followed existing best practices to maximize the accessibility, usability, and credibility of the intervention for a wide range of people, including those with lower levels of literacy or cognitive impairments [98]. Short sentences, lists, and visual formats were used, and the training interaction was tailored to each patient where appropriate.

#### Role of the Study Coordinator

The literature indicated that this study had the potential to generate negative feelings in the participants. The sources could be the wait, delay or expectation that their *match* would make contact, or difficulties encountered in dealing with the end of the study or the end of the relationship. Some organizations provide support throughout to volunteers face to face or by using technology while covering users from a wide age range [99]. Offered volunteer supervision has been described in the form of monthly multidisciplinary meetings, one-to-one supervision sessions, or telephone support [46,95-97,100]. In a previous study, when an intervention was remote, web-based support from a staff member and occasional telephone calls were found to be essential for participants to remain in the study and continue to use the digital intervention [101].

Therefore, it was decided that a study coordinator would be available for the participants, providing one-to-one access to support and supervision whenever the participants felt it was required in addition to proactively contacting all participants once per month. This would enable the provision of constant support to volunteers, who are community laypeople who could end up facing potentially unfamiliar situations that they may not know how to deal with (eg, managing patients’ behavior or difficulties with the relationship ending). Patients equally require support, although negative symptoms could make them less proactive in contacting the study coordinator. Therefore, the lead author carried an additional work phone. The number was given to all participants, and she could be reached at any time (ie, 24 hours a day and 7 days a week). The monthly contact would routinely be through an audio call; if the participants did not respond, a message would be sent asking for their availability. Although evidence suggests that reminders can improve engagement with digital interventions, there is as yet insufficient data to indicate what types of messages are most likely to promote adherence [102-104]. It also remains unclear how people will respond to these motivational messages. What one person sees as encouraging might demotivate another [105,106].

#### Monitoring Communication

To improve the understanding of the interactions between patients and volunteers, a practical decision was made to monitor the communication between each pair.

Although various members of the expert advisory group initially raised ethical questions about privacy, most subsequently concurred with this choice given that it offered an additional way to ensure that the content was appropriate and did not raise any safeguarding concerns.

The option of creating a technical *app* for communication monitoring was initially explored. After seeking quotations from different companies, it was established that a purpose-built app would be too costly. An existing app was identified that monitored the content of written communication (SMS text messages, WhatsApp messages, and emails) and the frequency and duration of audio and video calls. Through discussions with the expert advisory group, this app was purchased and used.

#### Monitoring Physical Activity Through Step Count

In line with the overall aim of the intervention to facilitate improvements in the participants’ mental and physical health by establishing informal communication about daily life and encouraging healthy lifestyles [107], physical activity changes were assessed in 2 ways. First, an existing app to monitor step count was installed on the smartphones. Using this, the participants could check their step count and gain awareness of their physical activity. In addition, to address potential issues of participants not always carrying the smartphone, the International Physical Activity Questionnaire [108] was administered both at the beginning and end of the study.

## Discussion

### Principal Findings

This study reports an innovative model of intervention development using a combined approach with the MRC framework and the person-based approach, which might be followed by other researchers developing interventions.

The Phone Pal logic model was rooted in the weak ties, behavioral activation, and self-determination theories and based on 11 guiding principles and 3 mechanisms of change (ie, engagement, role model, and self-efficacy).

The three core components linked to the logic model are (1) having a match, (2) communicating remotely, and (3) using a smartphone. Each of these components consists of parts and requires characteristics to shape its existence. The characterization of the intervention components is made up of components as well and comprises (1) volunteer characteristics, (2) patient characteristics, (3) role of the volunteer, (4) role of the patient, and (5) remote connection only through a smartphone.

For the intervention development, additional components of duration, frequency, methods, and content of communication were chosen. Finally, for the operationalization of the intervention, the following components were added: matching, training, providing a study coordinator, and monitoring the participants’ communication and physical activity.

### Strengths and Limitations

A main strength of this intervention development was its systematic process and consultation involving multiple methods and experts. This addresses the literature requirement for a greater focus on the developmental stage of interventions, allowing them to adapt on implementation [109,110]. It was also informed by relevant frameworks; that is, the MRC framework [28] and the person-based approach [29]. Both endorse the rigorous execution of intervention development and recommend the use of both quantitative and qualitative approaches, encouraging the lead author to combine existing evidence and use the most appropriate methods. This is of particular importance, given the increased awareness and encouragement of the publication of the development phase of interventions [111-114]. There is a notable gap in the literature relating to the systematic design of volunteer interventions despite the research conducted in this area.

Another advantage of this approach is that the intervention development involved participant perspectives throughout in the form of advisors to the research process and as study participants. This adheres to the literature recommendations that suggest that this process ensures the success of digital interventions [115,116]. Furthermore, both frameworks also emphasize the importance of collaborating with experts and clarifying how this involvement influenced the development process [117]. This logic model has been shaped following an extensive process of evidence collation and consultation with numerous stakeholders. It is also simple enough to capture the core elements necessary for this feasibility stage.

Although this approach has several strengths, it also has limitations.

The first limitation relates to the relatively linear process of intervention development in which one process informed the next (ie, a series of studies provided information for the logic model, which subsequently influenced the intervention development and operationalization).

A further potential problem with using the volunteer and patient advisory groups is that they may try to anticipate the needs of others, which they may not do well, rather than simply reporting their own experiences and views [29].

There are also potential challenges that may be faced when developing an intervention that targets different users simultaneously albeit with patients as the primary focus. Patients and volunteers vary in their characteristics. The inclusion criteria are necessarily dissimilar, and the opinions of the 2 parties may disagree on their vision of the intervention and, therefore, influence the other’s role or adherence. They may also have different beliefs about their own and others’ mental health condition or how they feel about being in contact with others. Not only may these views vary according to their role in the intervention but they may also reflect their individual characteristics (ie, age, cultural background, health condition, digital literacy, or previous experiences). To address this, the lead author spoke with a diverse range of potential users to ensure that she had insight into as many different and relevant perspectives as possible, thus enabling the intervention to be customized for all individuals.

Although the MRC framework is the most widely used approach to intervention development, it only provides a guideline for the relevant elements and the research questions to consider. It does not provide a prescriptive approach to development methodologies related to the varying contexts in which interventions can be used. In addition, there are challenges in incorporating the user perspective. The person-based approach is not intended to replace but to complement the well-known *theory-based* and *evidence-based* approaches that incorporate behavioral science into intervention development [28,118].

A potential limitation can be rooted in the intervention complexity (ie, there are numerous different types of interactions between components, a range of behaviors required by those delivering and receiving the intervention, varied targeted levels, a variety of outcomes, and a high level of flexibility and customization of the intervention [119]). It is challenging for a logic model to accurately capture this complexity. Logic models can be used to model complex interventions that adapt to context. However, it has been suggested that more flexible and dynamic models are required [27].

In addition, for clarity, the logic model portrays the main hypothesized pathways between the components, principles, mechanisms, and outcomes of the intervention. In reality, as previously acknowledged, it is likely that the intervention components operate on multiple principles and, therefore, multiple mechanisms.

Finally, as in other psychosocial interventions, the researchers had little control on how the intervention was delivered and would rely on the participants’ descriptions to assess its implementation.

### Implications for Future Research

The systematic process and theoretically sound strategy through which the intervention was developed enables other researchers to see clearly how the intervention was designed and why the components were selected in line with the contextual factors that were hypothesized as influential on the necessity and acceptability of the intervention. The logic model developed in this research is relevant to the particular contextual issues present in an intervention for people with psychosis based in London. However, this could provide future researchers with a platform for adapting this to translate to other populations and settings.

The Phone Pal logic model is hypothetical and depicts how core intervention components relate to the intervention principles and hypothesized mechanisms of change. It may not be possible to determine the exact components necessary or those that might have the most impact on the outcomes. Similarly, the mechanisms of change are not fully defined. This model might be built in future research to elucidate how and why any changes might be achieved. Future research should investigate which components contribute most or are essential to a successful intervention (eg, the remote contribution with the volunteer or having a smartphone), which components are superfluous, and how the components interact to influence outcomes [28]. Although this model recognizes the potential contextual factors affecting the acceptability and necessity of this intervention, it may be that in other contexts, different challenges may emerge, requiring the adaptation of this model and modification or substitution of the current components. Future research should explore how these components operate, which principles they promote, and the associated mechanisms. It may be plausible that other components could usefully contribute. Studies rarely specify whether the factors related to the delivery context affect which components are delivered, but the factors themselves are not always easy to capture. Whether these principles are useful targets for interventions for people with other conditions or in other settings is another topic for further investigation.

Previous logic models on volunteering [120,121] have hypothesized how volunteering may operate, focusing on the organization and requirements such as the acquisition of skills and retention of volunteers. In contrast, the logic model proposed in this study is the first to outline and place a focus on the hypothesized relationships between the patient and volunteer engaging in digital and remote communication. This may be helpful for the wider conceptualization of volunteering, distancing itself from the principle of the *organization* and focusing on person-to-person interactions. Previously, no specific model has been conceptualized that hypothesizes the relationships between patients and volunteers either face to face or digitally. This may inspire future research to potentially construct a model that focuses on face-to-face interactions.

The operationalization of this intervention could inform future research as it provides a clear map of the processes and outcomes of the development of a digital intervention using different components.

The main implication of this intervention development is allowing for further testing in a feasibility study before the final stages of the MRC framework—evaluation and implementation.

### Conclusions

The intervention development followed the MRC framework for developing complex interventions as well as the person-based approach. The intervention was developed following the evidence synthesis of a literature review, a focus group study, and a survey after consultation with advisory groups and input from a range of stakeholders, including patients, volunteers, clinicians, and academics. The developed logic model outlines the contextual factors, intervention, and short- and long-term outcomes. The operationalized intervention required matching 1 patient with 1 volunteer to communicate with each other through a smartphone via SMS text messages, WhatsApp messages, emails, and audio or video calls. All participants were encouraged to carry the smartphone throughout the day, engage in social and physical activities, and keep in contact with their usual friends. Each participant was encouraged to communicate with their match at least once per week for a 12-week period using informal conversation. The intervention provides guidance for the duration and frequency of communication (ie, once per week) but does not provide any recommendation regarding the different communication methods (ie, SMS text messages, WhatsApp messages, emails, and audio and video calls), leaving this decision completely to each patient-volunteer pair.

The findings of this study can inform future research. It presents the initial development phases of the intervention, the map of processes and outcomes from the logic model, and the actual operationalization. Practical issues faced could provide useful insights into the reality of preparing the operationalization of a digital intervention using multiple components.

## Appendix

Multimedia Appendix 1 Overview of the 4 stages of the intervention development process using an adapted version of the Medical Research Council framework and the person-based approach.

Multimedia Appendix 2 Smartphone provided during the study.

## Abbreviations

CONSORT: Consolidated Standards of Reporting Trials
MRC: Medical Research Council
REC: Research Ethics Committee
SMI: severe mental illness
