# Supplementary material for: An Intervention to Connect Patients With Psychosis and Volunteers via Smartphone (the Phone Pal): Development Study
Source: JMIR Form Res. 2022 Jun 2;6(6):e35086. doi: 10.2196/35086 (PMC9204578; doi:10.2196/35086)
Supplement: Multimedia Appendix 1 [file formative_v6i6e35086_app1.pdf]

Appendix 1. Overview of the four stages of the intervention development process using an adapted version of the MRC framework and the person-based approach

Intervention development stage	Aim	MRC recommendations	Person-based approach recommendations	Processes undertaken
**Early**	Identify the evidence base;<br><br>Identify theory of the intervention.	Examine relevant evidence;<br><br>Examine relevant theory;<br><br>Supplement with primary research with stakeholders (those targeted by or delivering intervention).		Evidence from the literature;<br><br>Survey of patients' preferences;<br><br>Focus groups study to elicit views on intended behavioural changes, previous experiences, barriers and facilitators.
**Early middle**	Identify key behavioural issues, needs and challenges;<br><br>Identify the guiding principles.	Consult with experts and stakeholders.	Focus groups study of views of volunteers and mental health professionals;<br><br>Create guiding principles comprising intervention objectives to address behavioural challenges and distinctive components to address objectives.	Create an outline of the intervention aims and objectives including the key components to achieve the objectives using:<br><br>- input from a multi-disciplinary team of academics and clinicians, and<br><br>- input from patients and volunteers.
**Late middle**	Design the intervention procedures.	Provide full description that can be replicated;<br><br>Consult with experts and stakeholders.		Interventions description.

**Late**	Operationalise the intervention procedures.		Elicit participant reactions to intervention and iteratively modify.	Patients' and volunteers' advisory groups;<br>Experts consultation and advisory group.
