# Supplementary figures and images for: An Intervention to Connect Patients With Psychosis and Volunteers via Smartphone (the Phone Pal): Development Study
Source: JMIR Form Res. 2022 Jun 2;6(6):e35086. doi: 10.2196/35086 (PMC9204578; doi:10.2196/35086)

## **Appendix 2.** Smart-phone provided during the study

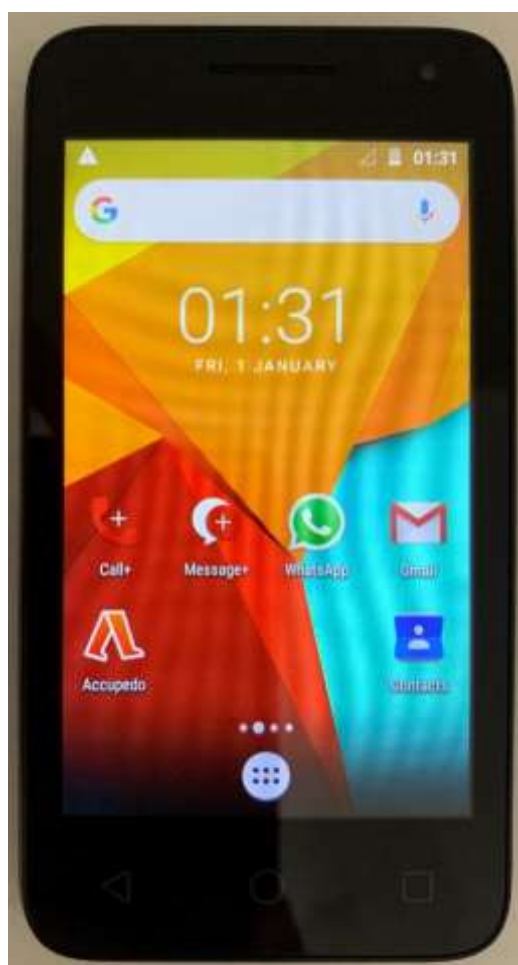

Supplement: Multimedia Appendix 2 [file formative_v6i6e35086_app2.pdf]
